# ^13^C MRI of hyperpolarized pyruvate at 120 µT

**DOI:** 10.1038/s41598-024-54770-x

**Published:** 2024-02-23

**Authors:** Nicolas Kempf, Rainer Körber, Markus Plaumann, Andrey N. Pravdivtsev, Jörn Engelmann, Johannes Boldt, Klaus Scheffler, Thomas Theis, Kai Buckenmaier

**Affiliations:** 1 High-Field Magnetic Resonance Center, Max Planck Institute for Biological Cybernetics, 72076 Tübingen, Germany; 2 Physikalisch-Technische Bundesanstalt, 10587 Berlin, Germany; 3 Institute for Molecular Biology and Medicinal Chemistry, Medical Faculty, Otto-von-Guericke University, 39120 Magdeburg, Germany; 4 Section Biomedical Imaging, Molecular Imaging North Competence Center (MOIN CC), Department of Radiology and Neuroradiology, University Medical Center, Kiel University, 24118 Kiel, Germany; 5 Departement of Biomedical Magnetic Resonance, Eberhard-Karls University, 72076 Tübingen, Germany; 6 Departement of Chemistry and Physics, NC State University, Raleigh, 27695 USA

**Keywords:** Magnetic resonance imaging, Biomarkers, Chemical physics, Applied physics

## Abstract

Nuclear spin hyperpolarization increases the sensitivity of magnetic resonance dramatically, enabling many new applications, including real-time metabolic imaging. Parahydrogen-based signal amplification by reversible exchange (SABRE) was employed to hyperpolarize [1-^13^C]pyruvate and demonstrate ^13^C imaging in situ at 120 µT, about twice Earth’s magnetic field, with two different signal amplification by reversible exchange variants: SABRE in shield enables alignment transfer to heteronuclei (SABRE-SHEATH), where hyperpolarization is transferred from parahydrogen to [1-^13^C]pyruvate at a magnetic field below 1 µT, and low-irradiation generates high tesla (LIGHT-SABRE), where hyperpolarization was prepared at 120 µT, avoiding magnetic field cycling. The 3-dimensional images of a phantom were obtained using a superconducting quantum interference device (SQUID) based magnetic field detector with submillimeter resolution. These ^13^C images demonstrate the feasibility of low-field ^13^C metabolic magnetic resonance imaging (MRI) of 50 mM [1-^13^C]pyruvate hyperpolarized by parahydrogen in reversible exchange imaged at about twice Earth’s magnetic field. Using thermal ^13^C polarization available at 120 µT, the same experiment would have taken about 300 billion years.

## The combination of hyperpolarization with sensitive magnetometry enables MRI at ultra-low magnetic fields

Ultralow-field magnetic resonance imaging (ULF-MRI) is an innovative approach to magnetic resonance imaging (MRI) that operates at low magnetic field strengths, typically below 10 mT^1–3^. Unlike high-field (HF) MRI, ULF-MRI does not require expensive, heavy, and bulky superconducting magnets. The challenge associated with ULF-MRI lies in (i) less induced voltage by precessing spins in normal pick-up coils at kHz frequencies, and (ii) low sensitivity caused by low spin polarization. Spin polarization refers to the alignment of nuclear spins in a determined direction, resulting in a net magnetic moment. Both of these challenges were addressed by (i) using a superconducting quantum interference device (SQUID) as a highly sensitive magnetometer for low-frequency detection^4,5^, and (ii) parahydrogen-based signal amplification by reversible exchange (SABRE). The most targeted hyperpolarization tracer for medical applications at present is pyruvate, which is being explored and established by Dynamic Nuclear Polarization (DNP) for cancer diagnostics.^6,7^ Imaging of 50 mM [1-^13^C]pyruvate was made possible by an enhancement with SABRE of 7.7 × 10^7^ when compared to thermal polarization at 120 μT. This corresponds to a polarization of about 0.8%, which reduces the acquisition time by a factor of 6 × 10^15^, since the acquisition time is proportional to the inverse square of the MR signal. In our case, the full 3D images presented below would have taken about 2.3 trillion years to acquire with thermal ^13^C polarization, whereas our hyperpolarized experiment lasted about 200 min.

In this work, a home-built ULF MRI system was based on SQUIDs due to their superior sensitivity and bandwidth over atomic magnetometers. SQUIDs have a rich history and are widely used in scientific research and it is precisely their exquisite sensitivity to magnetic flux in a wide frequency range down to zero frequency that makes them ideal sensors for zero field and ULF-MRI^8–11^. Such highly sensitive magnetometers make ULF-MRI a low-cost alternative to conventional high-field MRI (HF-MRI), with potential applications in medical diagnostics, neuroscience, and material science. Over the last decade, ULF-MRI has already provided promising results for imaging of soft tissues^2,12^, current density imaging^13–15^, detection of explosives in luggage^16^, and imaging in the vicinity of metals^17,18^. However, due to low thermal polarization, ULF-MRI cannot readily compete with HF-MRI in terms of image resolution and acquisition speed.

To overcome the limitation of low thermal polarization, primarily two approaches have been developed: Prepolarization^3,4^ and hyperpolarization (HP). Both methods have been used for ULF MRI experiments, including the following HP methods: Overhauser dynamic nuclear polarization (ODNP)^5,19,20^, spin-exchange optical pumping (SEOP)^21^, and parahydrogen (*p*-H_2_) -induced polarization (PHIP) techniques^22–25^. *p*-H_2_ as the nuclear spin ground state of hydrogen, is readily produced by cooling hydrogen gas e.g. in the presence of iron(III)-oxide-hydroxide (FeO(OH)) as catalyst. For example, at 25 K about 99% *p*-H_2_ content is obtained, which is conserved at room temperature in the absence of any (para-) magnetic material due to the kinetically hindered process for the transition to the higher energy ortho states. *p*-H_2_ represents an exceptionally highly ordered spin source, which can be converted into nuclear spin alignment on other molecules^26–28^.

This research work was based on a non-hydrogenative PHIP variant, which relies on reversible interactions of *p*-H_2_ with the substrate mediated by a transition metal catalyst. This approach was termed signal amplification by reversible exchange (SABRE) and has attracted much attention since its discovery, as SABRE has significantly expanded the scope of hyperpolarizable molecules^29^ and is simple to implement while being easily repeatable.

In both hydrogenative PHIP and non-hydrogenative SABRE, low magnetic fields can be used to transfer the spin order from *p*-H_2_ to other protons and heteronuclei^24^. The usage of a field-cycling ULF-MRI setup enabled the generation of polarization and in situ direct detection without sample shuttling. Furthermore, SABRE can provide continuous HP and therefore, two-dimensional nuclear magnetic resonance (NMR)^30^ or MRI^31,32^ are readily implemented at low and high magnetic fields. Even though literature data provides ample examples of two- and three-dimensional ^1^H images of phantoms and human subjects at ULF^2,18,32–36^, very few images of X-nuclei have been shown at ULF. One of those very rare examples includes hyperpolarized ^3^He imaging (2 mT)^21^.

We demonstrated ^13^C imaging inside of a SABRE hyperpolarization reactor. In the reactor, we hyperpolarized [1-^13^C]pyruvate using two different spin order transfer approaches for SABRE. The first method is called SABRE in shield enables alignment transfer to heteronuclei (SABRE-SHEATH), which employs small microtesla fields^37–39^, and the second is called low-irradiation generation of high tesla—SABRE (LIGHT-SABRE), which employs low-power radio frequency (RF) irradiation^40–43^. LIGHT-SABRE was previously used for in situ ^15^N HP and imaging at 9.4 T^31^. These techniques were recently proven to be useful for the preparation of HP for in vivo imaging using HF-MRI^44,45^. Here we demonstrated that both HP approaches are suitable for ^13^C ULF-MRI. After HP with either SABRE variant, the ^13^C images were acquired with a SQUID-based ULF-MRI system^5^ operating at 120 µT, detecting the ^13^C signals at 1.3 kHz. The *k*-space was encoded with two phase-encoding gradients and one read-out gradient to produce a 3D image of the SABRE hyperpolarized metabolite [1-^13^C]pyruvate.

## Results

### 3D-imaging setup with a SQUID

**Figure 1:**
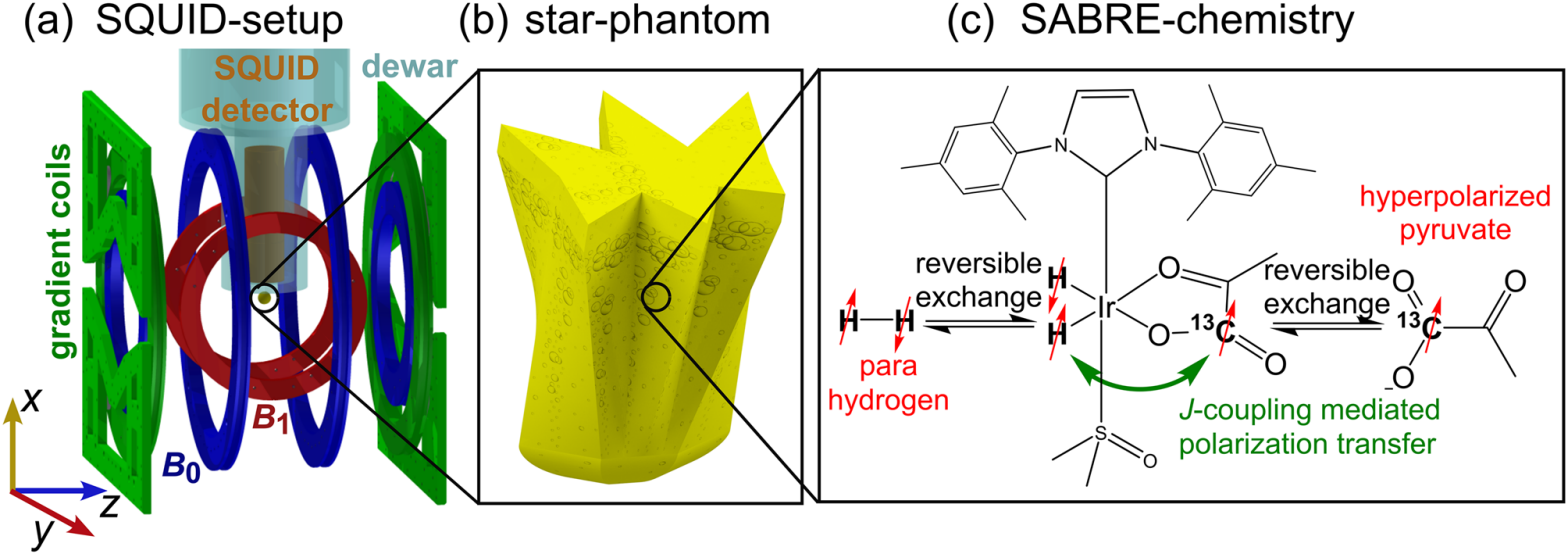
Overview of the SQUID ULF MRI setup with reaction chamber/imaging phantom and SABRE hyperpolarization scheme. (**a**) SQUID setup including gradient coils, dewar for liquid helium, SQUID, *B*_1_, and *B*_0_ coils. To keep the schematic clear, only the *x* gradient of the planar gradient coils is shown. (**b**) 3D rendered inner volume of the star-phantom of 15 × 15 × 20 mm, true size with illustrated parahydrogen bubbles. (**c**) Schematic of the SABRE process that yields hyperpolarization of the [1-^13^C]pyruvate substrate via *J*-coupling interactions in appropriate alternating (AC) or constant (DC) magnetic fields.

To achieve ^13^C ULF-MRI, we constructed a SQUID setup^5^ with MRI capabilities (Fig. 1a) and added a star-shaped reaction chamber where SABRE polarization can be created (Fig. 1b). The SQUID-based magnetic field detector was located in a low-noise liquid helium dewar, and was placed above the imaging phantom, which was centered in the *B*_0_, *B*_1_, and gradient coils (Fig. 1a). For the experiments, the *G*_*z*_ gradient, which superimposes the *B*_0_ magnetic field, is realized by a Maxwell coil, whereas the *G*_*x*_ and *G*_*y*_ gradients are realized by approximately linear magnetic field gradients generated by two planar coils. The entire system is surrounded by a chamber to shield electromagnetic interference consisting of two layers of μ-metal and one of aluminum. A detailed description of the system and the coil parameters can be found in reference ^5^.

### Different SABRE approaches to hyperpolarize a star-shaped phantom

The star-shaped sample reactor was made of 3D printed polypropylene—a chemically resistant material—and was cooled down to 5 °C (Fig. 1b and Fig. S2, see also details in Supplementary Materials). The geometry of the reactor was deliberately chosen to ensure favorable bubble conditions, allowing easy diffusion of *p*-H_2_ to all regions within the reaction volume. Different sizes of spikes on the star were implemented to allow for evaluation of the achieved resolution. The sample itself consisted of 50 mmol/L sodium [1-^13^C]pyruvate, 5 mmol/L [Ir(COD)(IMes)Cl] SABRE precatalyst (IMes = 1,3-bis(2,4,6-trimethylphenyl)-1,3-dihydro-2H-imidazol-2-ylidene, COD = 1,5-cyclooctadiene) synthesized according to the protocol in ^46^, and 18 mmol/L dimethylsulfoxide (DMSO) dissolved in non-deuterated methanol. The concentration of the named substances increased over time due to the evaporation of methanol during the several hours of the experiment.

During the experiment the SABRE catalyst provides a constant exchange of *p*-H_2_ and [1-^13^C]pyruvate (Fig. 1c), which, under suitable coupling conditions (SABRE-SHEATH or LIGHT-SABRE in our case), results in a hyperpolarization build-up on [1-^13^C]pyruvate. Both *p*-H_2_ and the substrate, [1-^13^C]pyruvate, are loosely bound to the Ir-complex, allowing reversible exchange. The SABRE-SHEATH and LIGHT-SABRE techniques rely on the *J*-coupling between *p*-H_2_ and the substrate that occurs at an energy level anti-crossing (LAC)^29,47^. For SABRE-SHEATH the *J*-coupling and the difference between the Larmor frequencies of the proton and the targeted heteronucleus must be of the same order to establish a LAC, which is typically established at magnetic fields below a few µT^37–39^. LIGHT-SABRE, on the other hand, uses an alternating magnetic field in the form of an RF field to establish a LAC condition and transfer polarization to X nuclei^40–43^. It has been demonstrated previously that [1-^13^C]pyruvate can be hyperpolarized with SABRE^48–51^ and detected with a SQUID at ULF^52^. This research work has now shown that [1-^13^C]pyruvate polarization can be sufficiently high for molecular imaging at 120 µT.

### Highly detailed ^13^C MRI at ultra-low magnetic field

We conducted a comparative study of ^13^C ULF-MRI, imaging [1-^13^C]pyruvate hyperpolarized through SABRE-SHEATH and LIGHT-SABRE techniques. The concentration of [1-^13^C]pyruvate amounted to 50 mmol/L, which correlates with 0.2% of the samples’ molecules. In the case of SABRE-SHEATH (see Methods section and Fig. 3a for details), the initial step involved the preparation of ^13^C polarization. Specifically, the sample was subjected to *p*-H_2_ bubbling for 15 s at a magnetic field strength (*B*_LAC_) of 0.35 µT and kept for additional 9 s at that field without bubbling (about 24 s total bubbling time). This resulted in a ^13^C polarization level of approximately 0.8%^52^. It is worth noting that the application of linear gradients at such low magnetic fields of below 1 µT proves to be challenging because the gradients also generate concomitant gradients exceeding the strength of the *B*_0_ field. Consequently, the *B*_0_ magnetic field was raised to ~ 120 µT, a selection also made to ensure that the MR signal frequency (~ 1.3 kHz for ^13^C) fell within a range of minimal sensor noise. Noise originating from current sources can couple with the SQUID detector via the magnetic field coils, even though the setup is housed in a chamber to shield electromagnetic interference. Accordingly, a trade-off between filtering and bandwidth of the current source had to be made, given the need for rapid changes in the static magnetic field during the field cycling experiments.

**Figure 2:**
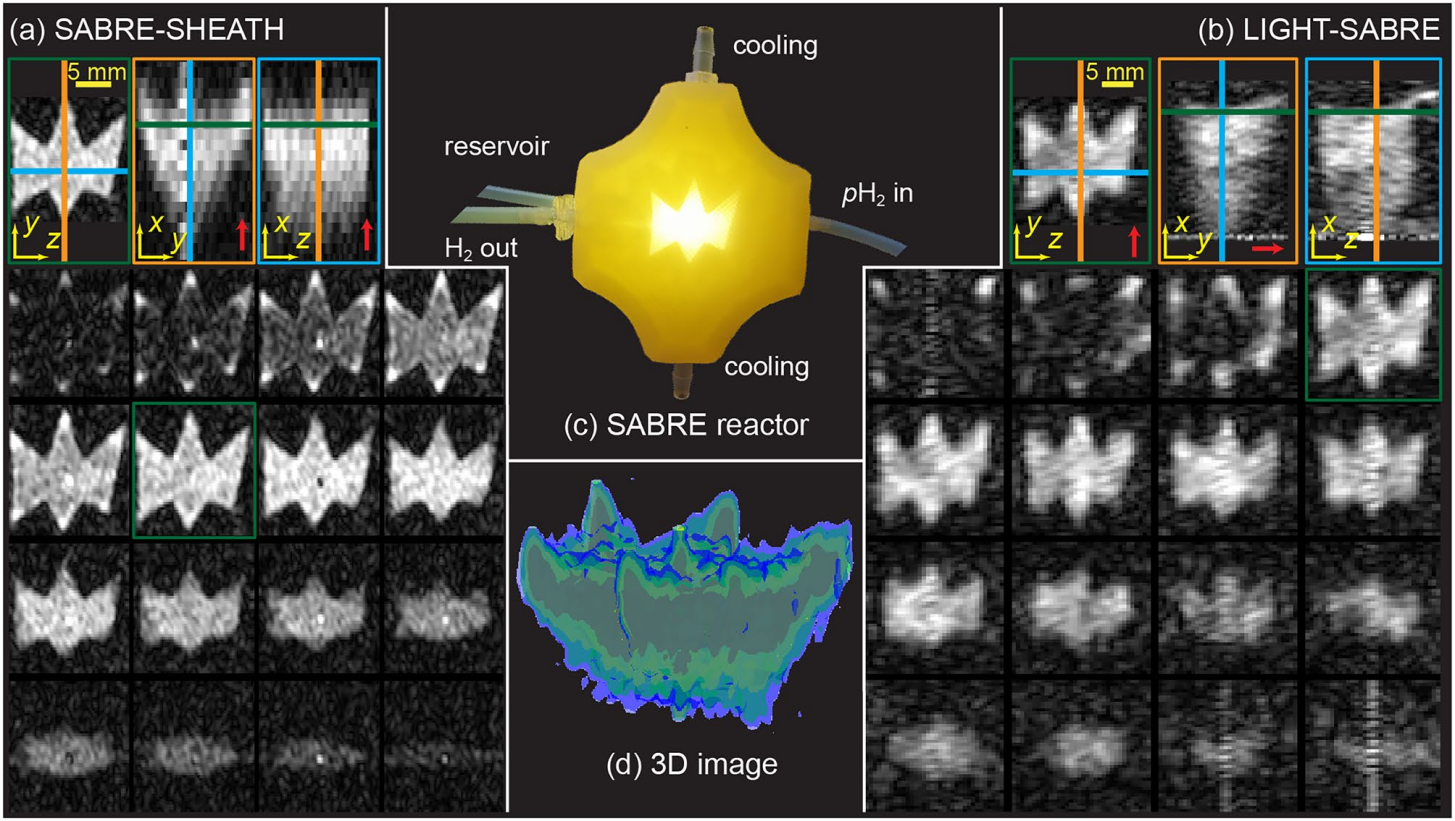
^13^C ULF MRI of hyperpolarized [1-^13^C]pyruvate in a SABRE reaction chamber. 3D spin-echo image of [1-^13^C]pyruvate polarized with SABRE-SHEATH (**a**) or LIGHT-SABRE (**b**) HP sequence. Red arrows indicate the frequency encoding direction. Photograph of 3D printed SABRE reaction chamber, illuminated from underneath (**c**). 3D multi-isosurfaces image acquired with SABRE-SHEATH (**d**).

**Figure 3:**
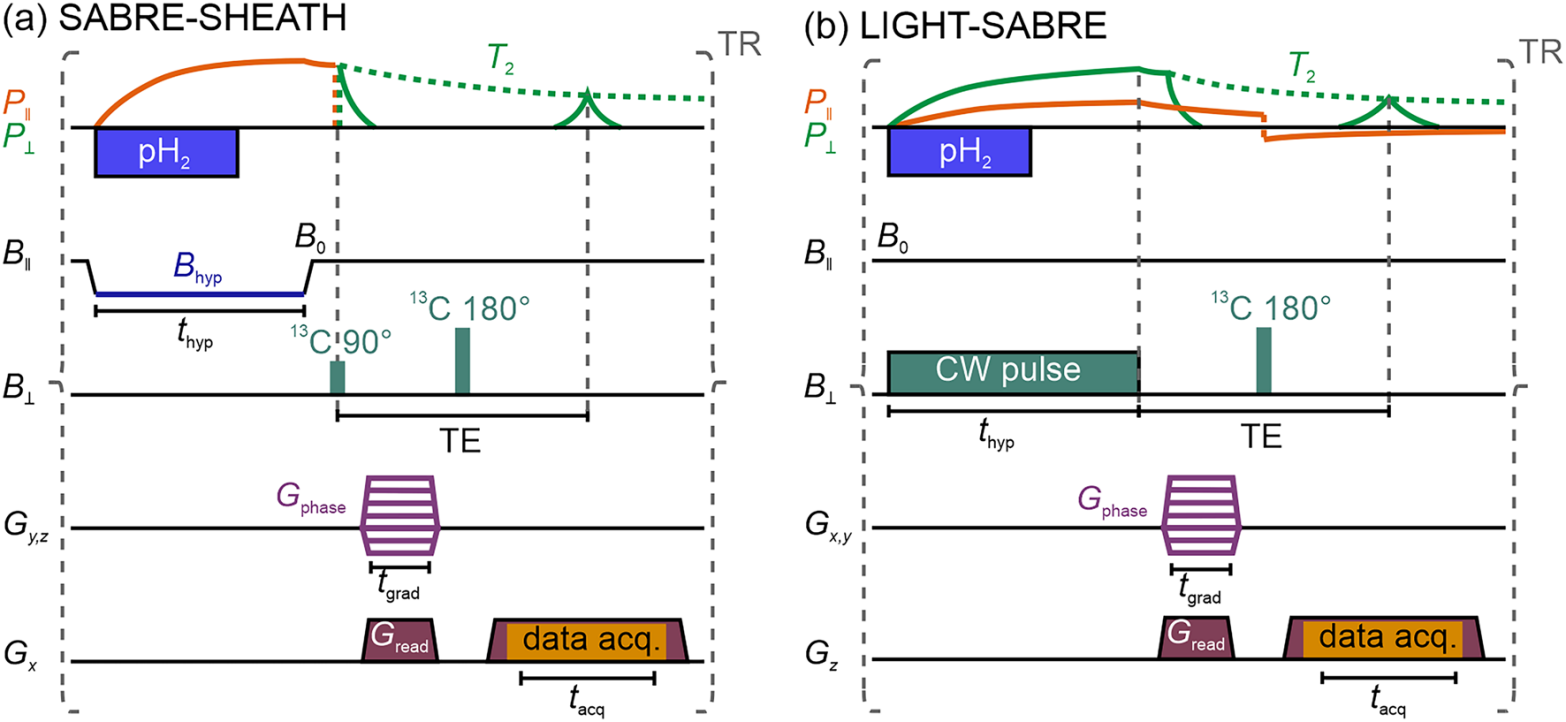
Schematics of the SABRE-SHEATH (**a**) and LIGHT SABRE (**b**) image sequences. In both cases a high spin order is initially produced through bubbling of *p*-H_2_. For SABRE-SHEATH (a), a field-cycling of *B*_‖_ to *B*_hyp_ ≈ *B*_LAC_ for polarization transfer is applied. The HP phase is followed by a ^13^C 90° pulse to flip the polarization in the transversal plane *P*_Ʇ_. In contrast, the hyperpolarization of the LIGHT-SABRE sequence (b) is based on a CW pulse in the transversal plane *B*_Ʇ,_ resulting in transversal polarization *P*_Ʇ_, obviating the need for a 90° pulse. Both hyperpolarization sequences are followed by two concurrent phase gradients encoding two spatial dimensions. A subsequent ^13^C 180° pulse refocuses the polarization to a maximum during the readout period, during which the third spatial dimension is encoded by a read gradient.

After the HP was established and the field was set to ~ 120 µT, a 3D spin-echo sequence with one frequency-encoding and two phase-encoding gradients was employed for image acquisition. Figure 2(a) highlights the obtained images of the star-shaped phantom. Since the relaxation times *T*_1_ and *T*_2_* of the ^13^C nuclei at this magnetic field strength are in the order of tens of seconds, and our echo time (TE) was 800 ms, the polarization decay during the imaging phase is negligible. This spin-echo sequence creates minor centerline artifacts due to imperfect 90° and 180° excitation pulses. The imperfection of the pulses is a result of *B*_0_ inhomogeneities leading to off-resonant pulses, the additional slight excitement of ^1^H nuclei due to a broad bandwidth of the pulses, and deviations emerging from the experimental determination of the angle of the *B*_1_ pulses. The centerline artifact appeared as a vertical line through the center of the image, which is particularly visible in the orange framed slice in Fig. 2(a) and the 3D rendered image in Fig. 2(d). The imaging time was about 221 min due to the need to acquire each line in *k*-space in two phase-encoding directions after the HP period. The reactor, depicted as a photo in Fig. 2(c), was resolved with a resolution of less than 1 mm in all directions in the MRI images. It is worth noting that the gradiometer of the SQUID system acts like a surface coil sitting on top of the reactor, therefore the signal intensity decreases with the distance from the sensor (top to bottom). In order to improve the resolution of the image, zero padding (zero filling) was used. Figure 3 shows the sequence schematics, the sequence parameters are listed in Tab. S1.

Further the LIGHT-SABRE scheme was implemented to overcome the challenges associated with field cycling at µT magnetic fields required for SABRE-SHEATH^52^. To prepare hyperpolarization with LIGHT-SABRE, the *B*_0_ field was held constant at 120 µT and a spin-locking *B*_1_ RF pulse was applied at the ^13^C Larmor frequency of 1.3 kHz for 20 s. By setting the *B*_1_ amplitude to approximately 1.1 µT and applying this field exactly on resonance, predominantly transverse magnetization is generated during the HP period, eliminating the need for a 90° pulse in the spin-echo sequence. A ^13^C polarization level of about 0.6% was attained in this LIGHT-SABRE mode^52^. To define the necessary parameter space for the experiment, we sped up the acquisition of a two-dimensional projection. As the reactor was higher than it was wide, it was possible to reduce the number of steps in *k-*space by changing the direction of the read gradient from *x* to *y*. In the acquisition scheme, the sign of the 180° pulses was alternated for each line in *k*-space to shift the center line artifact away from the image center, as seen in the orange and blue framed image in Fig. 2(b). The total acquisition time for the full 3D data in this LIGHT-SABRE mode was 247 min.

Although the polarization level was slightly reduced compared to the SABRE-SHEATH scheme due to the slower polarization build-up for LIGHT-SABRE (build-up time constant of 26 s compared to 16 s with SABRE-SHEATH)^52^ and the slightly shorter HP periods, all features of the polarization reactor were resolved.

## Discussion

This research demonstrated ^13^C MRI of hyperpolarized pyruvate under ULF conditions for the first time. SABRE stands out as a straightforward and fast hyperpolarization technique compared to the more established dissolution-DNP approach that is already under evaluation in clinical trials for molecular imaging in patients. In contrast, full biocompatibility is still being worked on for SABRE because of the need to remove the methanol solvent and the iridium-based polarization transfer catalyst. However recent advances appear promising in this regard^44,45,53^. At the current stage, the 3D reactor images can already be used to optimize SABRE experiments. In particular, the 3D ULF-MRI images can immediately inform the design of new hyperpolarization reactors such as two-phase designs^54^. In addition, the presented method already allows evaluation of the homogeneity of HP within the reactor, specifically whether *p*-H_2_ uniformly hyperpolarizes the substrate within the reactor volume. In principle, this can be done with ^1^H images as well, however, in-voxel signal annihilation can occur due to the contrast in ^1^H images stemming not only from the hyperpolarized substrate but also from the hyperpolarized orthohydrogen, produced during SABRE. The orthohydrogen’s direction of polarization is reversed^47^, as detailed in the Supplementary Materials (see Fig. S1). For example, our 3D-printed reactor was leakproof for liquids, but hydrogen could diffuse through the walls and fill unintended voids in the reactor, leading to ^1^H signal from outside the liquid volume. Moreover, orthohydrogen causes motion artifacts due to its high diffusion rate. These factors contribute to the higher quality of the ^13^C images, which are clearer and better defined than the ^1^H image shown in Fig. S1.

It should also be noted that the LIGHT-SABRE sequence can be further optimized. For example, *x*-polarization can be adiabatically rotated into *z*-polarization by linearly decreasing the *B*_1_ field^45^, potentially increasing signal intensity.

In the images presented, a simple spin echo sequence was used, in which the sample was hyperpolarized for each line in *k*-space. However, the imaging process can be accelerated significantly by using multiple spin echo sequences such as balanced steady-state free precession (bSSFP)^7^ or turbo spin echo (TSE)^55^, which would allow the acquisition of multiple *k*-space lines per HP period. Further acceleration of the imaging time can easily be expected due to the extended *T*_1_ and *T*_2_ relaxation times of ^13^C nuclei, which are particularly beneficial in the context of bSSFP and TSE. In addition, 2D single-shot (only one HP period) sequences appear as a viable option for implementing TSE or bSSFP sequences^7^, thus promising *p*-H_2_-based ULF MRI in vivo as a realistic future opportunity once biocompatibility concerns are fully addressed.

In summary, *p*-H_2_-based HP offers a cost-effective, highly scalable alternative to other hyperpolarization methods, that significantly benefits ULF MRI with trillion-fold time savings compared to using thermal polarization at these low fields. SABRE hyperpolarized ULF-MRI may provide a viable alternative to high-field MRI, which is why it opens up a new magnetic field regime, associated with much lower restrictions concerning implants^17,18^. Hyperpolarized ULF-MRI is particularly interesting in combination with Magnetoencephalography,^34^ also acquired with sensitive magnetometers including SQUIDS.

## Methods

### SABRE-SHEATH

Two different hyperpolarization sequences were used to acquire the 3D images at *B*_‖_ = *B*_0_ ≈ 120 µT. One sequence used the SABRE-SHEATH hyperpolarization phase (Fig. 3a). In this case, the magnetic field parallel to the polarization of the sample *B*_‖_ was reduced to the *B*_LAC_ condition (≈ 0.35 µT) and *p*-H_2_ was fed into the reactor by bubbling for *t*_bubble_ = 15 s at a flow rate of 2 L/h. After the bubbling stopped, *B*_‖_ was held at *B*_LAC_ for another 8.4 s in order to stop the liquid flow caused by *p*-H_2_ bubbling and to prevent motion artifacts. The total hyperpolarization time *t*_hyp_ for this sequence was 23.4 s. It is important to note that SABRE-SHEATH generates longitudinal polarization *P*_‖_ as is the case in conventional MRI. The 3D readout was obtained by acquiring a *k*-space line with a single spin echo readout (echo time TE = 800 ms, *t*_acq_ = 1 s, repetition time TR = 24.9 s) after each hyperpolarization phase. Two phase encoding gradients *G*_phase_ of length *t*_grad_ and the readout gradient *G*_read_ with a maximum gradient strength of 1 mT/m were applied between the 90° and 180° *B*_1_ pulses of the spin echo sequence. The readout gradient was also maintained during data acquisition (Tab. S1).

### LIGHT-SABRE

The other sequence used the LIGHT-SABRE hyperpolarization scheme (Fig. 3b)^52,56^. Similar to the previous sequence, *p*-H_2_ was bubbled through the reactor for *t*_bubble_ = 12.5 s. However, instead of field cycling, a LIGHT or spin-lock induced crossing (SLIC) continuous wave (CW) *B*_1_ pulse with an amplitude of 1.1 µT and a frequency of 1.3 kHz, the ^13^C Larmor frequency at 120 µT, was applied. Following the *p*-H_2_ bubbling, the continuous wave pulse was extended for an additional 7.5 s, resulting in a total hyperpolarization phase of 20 s. Unlike the SABRE-SHEATH scheme, LIGHT-SABRE generates predominantly transverse polarization *P*_Ʇ_, making the 90° pulse of the spin echo sequence unnecessary. Instead, the two phase and the frequency encoding gradients were applied immediately after the hyperpolarization phase, followed by the 180° refocusing pulse and the data acquisition (TE = 800 ms, *t*_acq_ = 1 s, TR = 21.5 s).

### Supplementary Information

Supplementary Information 1.

Supplementary Information 2.

## Data Availability

All data necessary to understand and interpret the results is included either in the manuscript itself or in the supplementary materials. The depicted images’ raw *k*-space data and the Matlab scripts used for image reconstruction are available on request from the corresponding author and can be used for any non-commercial purpose.
