# Supplementary material for: 13C MRI of hyperpolarized pyruvate at 120 µT
Source: Sci Rep. 2024 Feb 23;14:4468. doi: 10.1038/s41598-024-54770-x (PMC10891046; doi:10.1038/s41598-024-54770-x)
Supplement: Supplementary file 1 — Supplementary Information 1. [file 41598_2024_54770_MOESM1_ESM.pdf]

Supplementary Materials for

^{13}C MRI of hyperpolarized pyruvate at 120 μT

Nicolas Kempf, Rainer Körber, Markus Plaumann, Andrey N. Pravdivtsev, Jörn Engelmann, Johannes Boldt, Klaus Scheffler, Thomas Theis, Kai Buckenmaier[1*]

Corresponding author: kai.buckenmaier@tuebingen.mpg.de

The PDF file includes:

Supplementary Text
Figs. S1 to S2
Table S1

Other Supplementary Materials for this manuscript include the following:

Data S1

3-dimensional ^{1}H images of a star-shaped SABRE reactor

The 3D imaging sequence for ^{1}H was very similar to the SABRE-SHEATH sequence [Fig. S1(a)]. However, one key difference was evident: the decrease in $B_{\|}$ below 1 μT was replaced by a ramp to $B_{hyp} \approx 6$ mT to facilitate polarization transfer from pH_2 to pyruvate. Additionally, the phase steps were reduced in general due to a lower signal intensity to achieve a sufficient SNR for the ^{1}H image through larger voxels. Under this conventional SABRE configuration, polarization was predominantly transferred to protons of the pyruvate. In addition, the gradient intensity was reduced by approximately a factor of 4 due to the higher gyromagnetic ratio of the protons.

The 3D images of ^{1}H [Fig. S1(b)] showed less clarity compared to the ^{13}C images. Especially in the upper sections of the reactor, signal annihilation was observed between pyruvate and orthohydrogen due to their opposite signs of hyperpolarization (during the SABRE reaction, MR invisible p-H_2 transforms into MR visible orthohydrogen). This phenomenon was most pronounced in the center of the reactor, coinciding with hydrogen bubbling activity [yellow ellipse in Fig. S1(b)]. In addition, an artifact manifested itself in the center of the image. In particular, a significant amount of signal was detected outside the reactor volume, possibly due to movement or diffusion of orthohydrogen through the reactor walls, filling voids created during the reactor printing process [green ellipse in Fig. S1(b)].

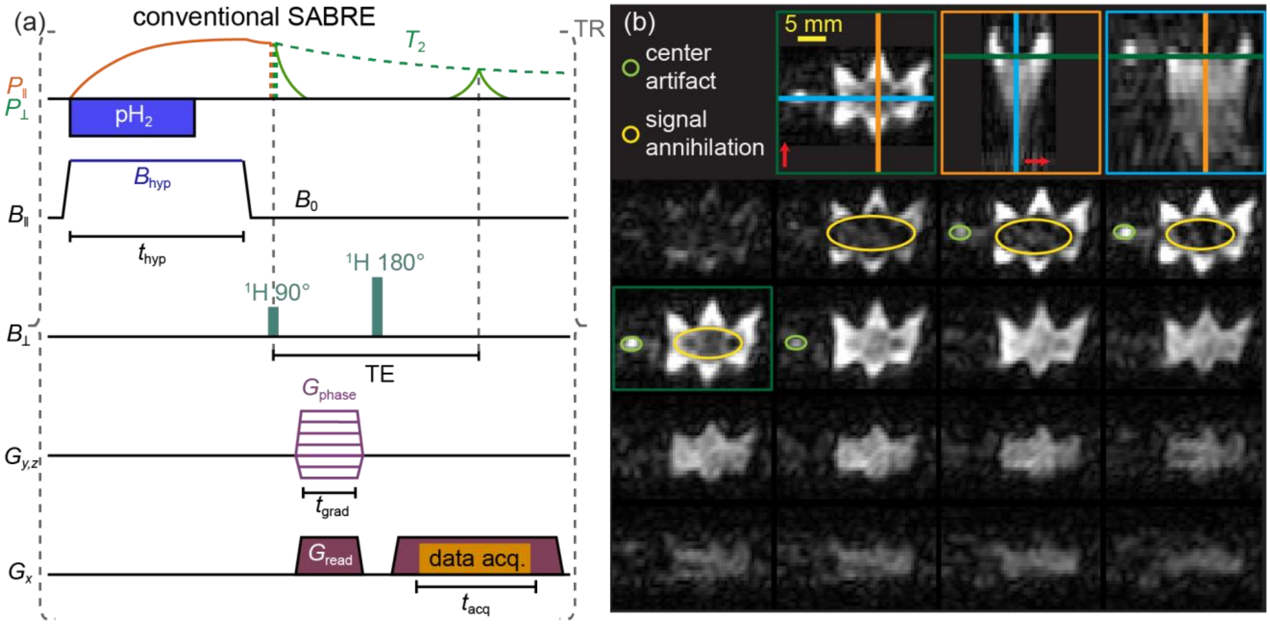

Fig. S1.

Schematic of the conventional SABRE sequence (a) and ^{1}H MR images (b) for the star shaped SABRE reactor, red arrows indicate the frequency encoding direction.

Reactor description and construction

The design of the reactor was chosen to be 3D-printable and to provide spatial orientation as well as an estimate of imaging resolution. Therefore, the sample holding volume (SHV) is a rejuvenating cylinder with a star-like shape as footprint. Each jag of the star-shape is 1 mm shorter than the one next to it. Polypropylene was chosen as the print filament because of its high chemical resistance. A second, toroidal chamber surrounds the SHV to provide temperature control by exchanging heat with a control fluid. The temperature was monitored by a fiber-optic sensor (Osensa PRB-100), placed in the temperature control fluid at the outlet of the reactor.

An always filled SHV was achieved by adding a reservoir. Within the reservoir the expelled liquid and gas mixture is separated, thus allowing continuous measurement for about 10 hours by recycling the sample.

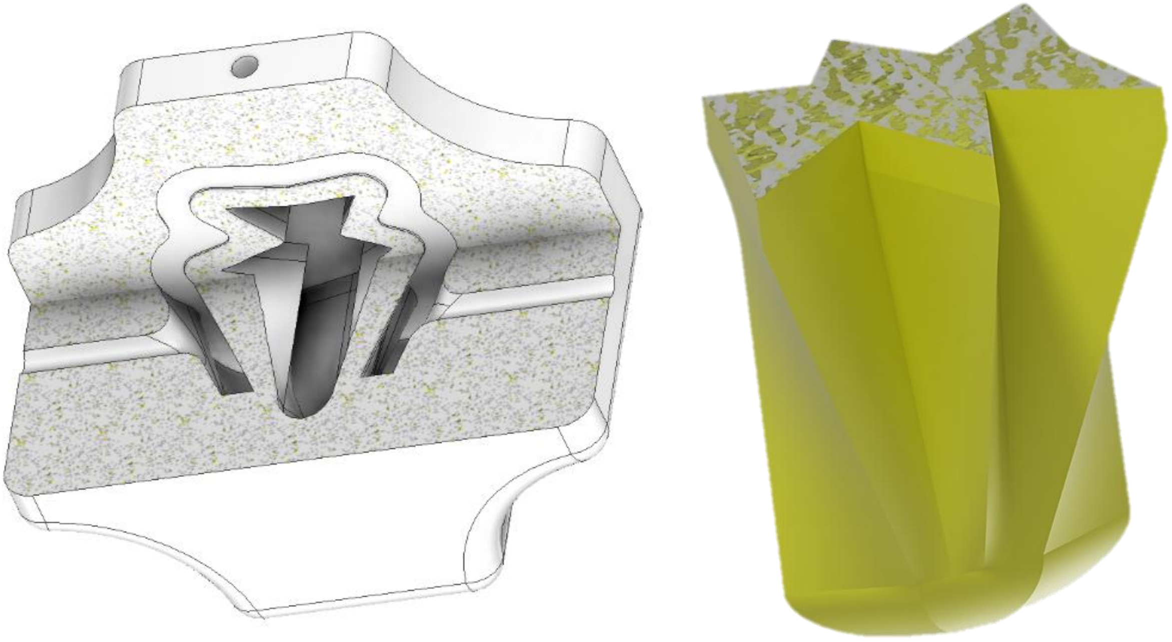

Fig. S2.

Cross section of the CAD file of the reactor with visible sample holding volume surrounded by the cooling chamber (left). Schematic of the sample holding volume (right).

Sample preparation

After measuring out the amount of each substance, 18 mmol/L DMSO was added to the non-deuterated methanol and placed in a sonic bath for 10 minutes to remove solved gases. This procedure yields empirically more polarization, probably because the Ir-precatalyst stays free of contaminations residing in the liquids. Subsequently the 50 mmol/L [1-^{13}C]pyruvate and 5 mmol/L Ir-precatalyst are added to the solution and placed again in the sonic bath for easy and optimal dissolution. The now homogenous solution was filled in a syringe and injected to the reservoir where it was immediately exposed to parahydrogen and cooled down to 5°C before the experiment.

Table S1.

Sequence parameters of the presented images

	LIGHT-SABRE (^{13}C)	SABRE-SHEATH (^{13}C)	Conventional SABRE (^{1}H)
B_0 [µT]	118.60	121.13	118.72
B_{hyp} [µT]	118.60	0.35	5860
t_{hyp} [s]	20	23.4	6
t_{bubble} [s]	12.5	15	2
t_{grad} [ms]	320	320	320
t_{acq} [s]	1	1	1
TE [ms]	800	800	800
TR [s]	21.5	24.9	7.5
Phase steps	16 x 43	19 x 28	13 x 22
Read direction	*y*	*x*	*y*
Imaging time [min]	247	221	36

Data S1. (separate file)
Rendered isosurfaces of signal intensity of the 3-dimensional SABRE-SHEATH image
