# Supplementary material for: 13C MRI of hyperpolarized pyruvate at 120 µT
Source: Sci Rep. 2024 Feb 23;14:4468. doi: 10.1038/s41598-024-54770-x (PMC10891046; doi:10.1038/s41598-024-54770-x)
Supplement: Supplementary file 2 — Supplementary Information 2. [file 41598_2024_54770_MOESM2_ESM.zip › 3D render/figure.xhtml]

13C image, SABRE-SHEATH


# Interactive threedimensional reconstruction of the 13C image obtained with SABRE-SHEATH

Isosurfaces of signal intensity [a.u.] where each color represents a different signal intensity.  Highest signal intensity. Lowest signal intensity.
